# Effect of Calcitriol on Bone Turnover and Osteocalcin in Recent-Onset Type 1 Diabetes

**DOI:** 10.1371/journal.pone.0056488

**Published:** 2013-02-20

**Authors:** Nicola Napoli, Rocky Strollo, Dario Pitocco, Carla Bizzarri, Ernesto Maddaloni, Daria Maggi, Silvia Manfrini, Ann Schwartz, Paolo Pozzilli, on behalf of I.M.D.I.A.B. Group

**Affiliations:** 1 Department of Endocrinology and Diabetes, University Campus Bio-Medico, Rome, Italy; 2 Division of Bone and Mineral Diseases, Washington University in St Louis, St Louis, Missouri, United States of America; 3 Bone & Joint Research Unit, William Harvey Research Institute, Barts and The London School of Medicine, Queen Mary, University of London, London, United Kingdom; 4 Diabetology, Catholic University, Rome, Italy; 5 Endocrinology and Diabetes, Bambino Gesù Children’s Hospital, Rome, Italy; 6 University of California San Francisco, San Francisco, California, United States of America; 7 Centre for Diabetes, The Blizard Building, Barts and The London School of Medicine, Queen Mary, University of London, London, United Kingdom; University of Michigan Medical School, United States of America

## Abstract

**Background:**

Vitamin D supplementation in childhood improves the achievement of peak bone mass. We investigated the effect of supplementation with calcitriol on bone turnover in recent-onset type 1 diabetes (T1D). Moreover, the association between osteocalcin and parameters of β-cell function and metabolic control was examined.

**Methodology/Principal Findings:**

We conducted a post-hoc analysis of a double-blind, placebo-controlled study of calcitriol supplementation to preserve β-cell function. 27 recent-onset T1D subjects, mean age 22 years, were randomized to 0.25 µg calcitriol per day or placebo (1∶1) and followed up for one year. Changes in bone formation (osteoclacin) and resorption (beta-CrossLaps) markers, and differences between placebo and calcitriol-treated group were evaluated. *At baseline*, osteocalcin levels were significantly lower in female than in male patients (*P*<0.01) while no other metabolic parameters as HbA1c and C-peptide differed between gender. No significant correlations were found in relation to HbA1c, insulin requirement and C-peptide. *At 1 year follow-up,* no significant differences were observed between calcitriol and placebo groups for osteocalcin and β-CrossLaps. In the placebo group osteocalcin levels were unrelated with parameters of metabolic control, such as C-peptide, insulin requirement or HbA1c. Changes of C-peptide, insulin requirement and HbA1c were not related to osteocalcin levels.

**Conclusions:**

Supplementation with 0.25 µg calcitriol per day to patients with new-onset T1D does not affect circulating markers of bone turnover. OC levels were unrelated to β-cell function and other metabolic parameters suggesting that OC is ineffective to control pancreatic function in presence of aggressive autoimmune destruction.

## Introduction

In 1948 Albright et al. investigated bone development in diabetic children linking for the first time diabetes with bone mass loss [1]. Since then several studies have examined the relationship between type 1 diabetes (T1D) and skeletal disorders showing an impaired peak of bone mass and, consequently, an increased risk of osteoporosis and fractures [2], [3]. A large variety of mechanisms have been proposed to explain this finding, (such as hyperglycemia, inflammation and generation of advanced-glycation end products into the bone matrix), but most studies have focused on the lack of anabolic effect of insulin on osteoblasts, indicating T1D as a condition of low bone turnover, meaning both osteoblast and osteoclast functions are suppressed [4], [5], [6].

Vitamin D deficiency has been associated with T1D [7] and several studies suggest that vitamin D may exert immunomodulatory effects. [7], [8]. At high doses calcitriol prevents insulitis and the development of experimental diabetes, by acting on the defective suppressor cellular function, or cytokine expression modulation [7], [9]. Moreover, supplementation with vitamin D during early childhood may decrease the risk of developing T1D [10], [11]. Contrary to what was expected, we have found that calcitriol supplementation is not effective in improving β-cell function or reducing insulin requirement in patients with newly diagnosed T1D [12].

However, a large amount of data suggests the importance of vitamin D to preserve bone accrual in young subjects [13], [14]. Vitamin D supplementation in deficient children and adolescents restores mineralization and may improve bone health in deficient children and adolescents [15]. The beneficial effect of vitamin D is even more pronounced when supplementation starts in infancy or at the beginning of the adolescence [16], [17], and seems to be primarily dependent on a reduction of bone resorption [18]. The achievement of peak of bone mass plays a major role in determining bone health throughout life. A 10% rise in bone mass during adolescence or early adulthood can halve the risk of an osteoporotic fracture in older age [19].

Nevertheless, according to our previous study, vitamin D deficiency is common in young T1D patients [20], a condition that may contribute to lower BMD and increases fracture risk [21], [22]. While several evidences support the efficacy of vitamin D supplementation at any age, no data are available in T1D patients in terms of bone turnover and fracture prevention.

In addition, new research lines link bone metabolism to pancreatic function. Thus, it has been shown that osteocalcin (OC), an osteoblast-derived protein, has endocrine effects acting on islet cells by stimulating β-cell proliferation and insulin secretion [23], [24]. As an insulin secretagogue, OC stimulates the expression of insulin genes [24], [25]. These findings have been partially confirmed in humans, showing an inverse correlation between OC and glucose homeostasis [26]. However, no data are available in young T1D patients on OC and its effect on parameters of β-cell function.

Therefore, we conducted a post-hoc analysis using sera collected in a previous clinical trial, aiming to investigate the effect of 1 year calcitriol administration on β-cell function [12], on another parameter, the bone turnover. Moreover, we also tested in the same patients the relationship between OC and β-cell function in patients with recent onset of T1D.

## Materials and Methods

### Ethic Statement

This study was approved by the Ethical Committee at University Campus Bio-Medico within the framework of the IMDIAB investigators type 1 study, with informed consent signed by patients or parents, where appropriate.

### Patients

A post-hoc analysis was conducted using clinical data and serum samples from a previous study [12]. Patients’, clinical management, randomization strategy and guidelines for insulin therapy were already published elsewhere [12]. Briefly, 27 patients with recent onset T1D were recruited in the metropolitan area of Rome by participating centers of the IMDIAB group. Patients’ inclusion criteria included: (a) age at presentation between 10 and 35 years; (b) duration of clinical disease (since the beginning of insulin therapy) <12 weeks; c) baseline C-peptide >0.25 nmol/l. In all patients, presence of autoantibodies to glutamic acid decarboxylase (GAD) confirmed diagnosis of T1D. After baseline measurements were completed and patients were blindly randomized to receive either calcitriol (0.25 µg/daily) or placebo and followed up for 1 year. All patients were treated with three injections of regular insulin at meal times and one of basal insulin at bed time.

### Evaluation of Bone Turnover

This was evaluated analyzing a marker of bone formation (osteocalcin) and a marker of bone resorption (serum β-CrossLaps). Levels of osteocalcin and serum β-CrossLaps were measured by EIA commercially available (ALPCO, USA). Serum 25-hydroxyvitamin D (25OHD) was measured by radioimmunoassay (Diasorin).

### Pancreatic Function

Fasting plasma C-peptide was evaluated to establish the degree of residual β-cell function and measured after euglycaemia was achieved by chemiluminescence on ADVIA Centaur analyzer, a two-site sandwich immunoassays using direct chemiluminescent technology. The reference range of fasting C-peptide established in 150 control subjects (71 females and 79 males matched for age and with no family history of T1D) was 0.1–1.3 nmol/l with intra- and inter-assay coefficient of variability between 10 and 15%, respectively.

### Statistical Analysis

Results are expressed as the mean ± SE and *P*<0.05 was considered statistically significant. The association between clinical variables with parameters of bone turnover and vitamin D were evaluated by simple correlation. Differences between means of the two groups were compared by unpaired t-test and non-parametric Mann–Whitney U-test, where applicable. Multiple comparison by ANOVA was used to compare treatment groups across time points. Data were analysed using SPSS (Chicago, IL, USA).

## Results

We analyzed bone turnover in 27 T1D patients (<12 weeks duration). Clinical features of patients are reported in Table 1.

**Table 1:** Clinical and biochemical features of the study population at baseline.

	Placebo group (n = 15)	Calcitriol group (n = 12)
Males [n(%)]	8 (53)	8 (66)
Age [yrs]	22.00±2.34	22.83±2.14
BMI [kg/m^2^]	22.07±0.62	22.56±1.53
C-peptide [nmol/l]	0.33±0.47	0.44±0.71
HbA1c [%]	8.56±0.38	9.60±0.90
Insulin Requirement [U/Kg/die]	0.40±0.03	0.42±0.03
25OHD [ng/ml]	26.78±3.80	24.02±2.61

Data are mean ± SE.

**Figure 1:**
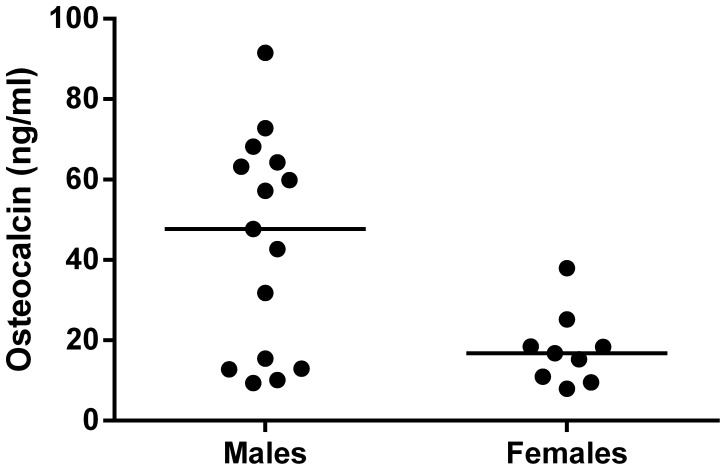
Osteocalcin levels at baseline according to gender. Osteocalcin levels were significantly higher in males than females (P<0.01).

**Figure 2:**
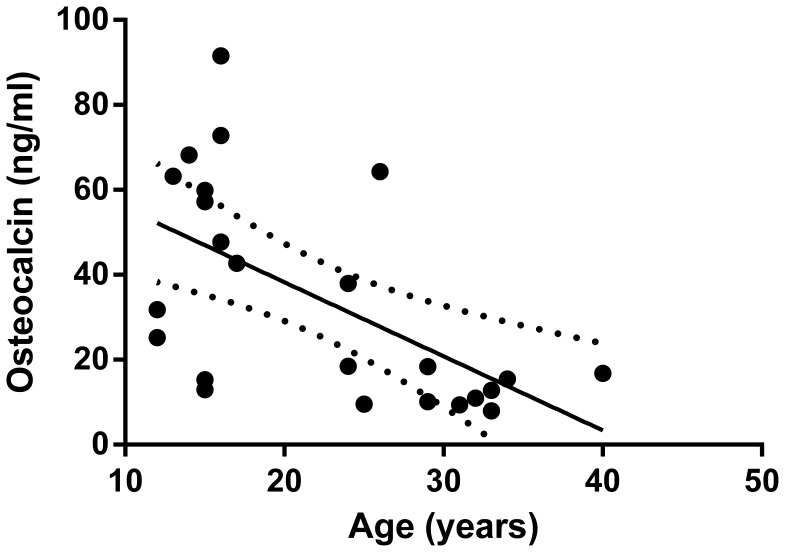
Correlation between osteocalcin levels and age in type 1 diabetes subjects at diagnosis. Baseline OC levels were negatively correlated to age (r = −0.59, *P* = 0.02). Dashed curves denote upper and lower 95% confidence intervals.

*At baseline*, no significant differences were found in terms of age and BMI between males and females. OC levels were significantly lower in female than in male patients (17.8±3.1 ng/ml vs. 43.9±6.9 ng/ml; *P*<0.01. Figure 1) while no other metabolic parameters as HbA1c, C-peptide differed between genders. Analyzing the whole group of patients, baseline OC levels were negatively correlated to age (r = −0.59, *P* = 0.02. Figure 2) while no significant correlations were found in relation to HbA1c, C-peptide or insulin requirement. BMI was positively correlated to age (r = 0.44, *P*<0.05) and to C-peptide (r = 0.58, *P*<0.001).

*At 1 year follow-up,* OC and β-CrossLaps levels did not change significantly compared to diagnosis nor in the placebo not in the calcitriol-treated group. No significant differences were observed between calcitriol and placebo groups for OC (37.2±11.8 ng/ml vs 25.8±7.4 ng/ml; *P* = 0.39) and β-CrossLaps (0.33±0.07 ng/ml vs 0.50±0.12 ng/ml; *P* = 0.29) (table 2).

**Table 2:** Biochemical markers of bone turnover during the trial.

	Baseline		12 months	
	Placebo group	Calcitriol group	Placebo group	Calcitriol group
Osteocalcin [ng/ml]	34.69±7.04	33.49±7.91	25.8±7.35	37.19±11.77
β-CrossLaps [ng/ml]	0.77±0.14	0.55±0.17	0.50±0.12	0.34±0.08

Data are mean ± SE.

No significant correlations were found at 12 months in the placebo group between OC and parameters of metabolic control, such as HbA1c, C-peptide or insulin requirement. Changes in any metabolic parameters across time were unrelated to OC levels.

## Discussion

The natural history of diabetes is related to long term complications that impact life quality and expectancy. The skeleton is an important target of diabetes complications and T1D patients exhibit bone loss and increased risk of fracture compared with healthy age-matched subjects [2]. Because T1D typically occurs in young age, the disruption in bone homeostasis affects bone accrual and peak of bone mass. A metanalysis confirmed the deleterious impact of T1D on bone health, showing a lower Z-score at lumbar spine and at proximal femur in patients with T1D compared to healthy subjects and consequent increased risk of hip fracture (RR = 6.94) [3].

The potential effect of calcitriol on bone turnover in T1D has not been fully evaluated. Administration of pharmacological doses of calcitriol in NOD mice showed a near two-fold increase of osteocalcin levels [9]. According to our knowledge, this is the first study assessing the effect of calcitriol supplementation on bone turnover in T1D patients. In this study 12 month intervention with the active form of vitamin D was not effective in modifying bone turnover markers.

To date, there are no available trials investigating the effect of calcitriol on bone metabolism in new diagnosed T1D young patients. Intervention studies conducted in adults and post-menopausal women have yielded variable results, ranging from no effective to a reduction of bone turnover [27]. Contrasting findings have been reported also by studies with vitamin D_3_, especially in young people and adolescents. Schou et al. have shown that vitamin D supplementation in healthy children did not affect bone turnover significantly [28]. Conversely, another study on young subjects treated with D_3_ showed that, similarly to an antiresorptive agent, vitamin D lowers primarily bone resorption [18].

This is also the first study to evaluate the association between OC levels and pancreatic function in T1D patients. Although limited by the small sample size, our results might suggest that metabolic action of OC is not relevant for controlling β-cell function in T1D. To date, few human studies explored the relationship between OC and glucose homeostasis and most of these were conducted in healthy subjects or in patients with type 2 diabetes (T2D) and limited by the cross-sectional design. OC levels have been reported to be lower in T2D compared to healthy subjects [29], inversely related to body mass index, fat mass, and plasma glucose [30], [31], [32], [33]. Our data follow a different trend, showing a lack of association between OC and β-cell function. Although human studies in patients with T2D and in animals support a positive feedback between osteoblast and β-cells, we may speculate that in a condition of continuous autoimmune damage against β-cells such as in T1D, the OC may be uneffective on controlling β-cell function. The relationship between OC and metabolic control in T1D subjects has been also investigated in a recent cross-sectional study by Thrailkill et al. [34]. In contrast to our findings, Thraikill et al. reported on a positive effect of OC on endogenous insulin production (assessed by authors as C-peptide/glucose ratio) [34], but several differences in term of sample size, population (new-onset vs. long-standing diabetes), design of the study (longitudinal vs. cross-sectional) and measure of endogenous insulin production (C-peptide vs. C-peptide/glucose ratio) limit the comparison between our study and the one conducted by Thraikill. In our study, OC levels were lower in females than males. This difference has been shown in healthy subjects, as a consequence of the effect of sex hormones on skeletal tissues during late-pubertal and post-pubertal stages [35], [36]. Other studies reproduced this difference also among young patients with T1D [37], [38]. Although OC levels are physiologically lower in females than males, we cannot exclude that diabetes-induced osteoblast disruption might amplify the difference we reported [39].

The significance of our findings may be limited by the lack of BMD measurements and the under-carboxylated osteocalcin (ucOC) dosage. However, a large body of clinical evidence suggests that total OC correlates to glucose homeostasis [26] similarly to ucOC [31], [40]. Moreover, although we did not measure serum levels of calcitirol, the same dosage was used by Walter et al. who shown significant raise of calcitiol serum concentrations in T1D after 9 months of treatment [41]. Finally, given the limited number of studied subjects and the *post-hoc* nature of the study, we cannot definitely rule out from this study the interplay between OC and pancreatic function in T1D patients and further studies, in larger cohorts of patients, are required.

In conclusion, the results of this study indicate that supplementation with 0.25ug calcitriol per day to patients with new-onset T1D does not affect circulating markers of bone turnover. However, larger ad-hoc trials with vitamin D investigating bone turnover and fracture outcomes in T1D are needed. OC levels were unrelated to β-cell function and other metabolic parameters suggesting that OC is ineffective to control pancreatic function in presence of aggressive autoimmune destruction. To disentangle the role of OC in T1D more studies are needed, with larger cohorts and longer follow-up.
